# Accelerated 3D self-gated cardiac cine imaging at 3T using a tiny golden angle and compressed sensing

**DOI:** 10.1186/1532-429X-18-S1-P305

**Published:** 2016-01-27

**Authors:** Xiaoyong Zhang, Guoxi Xie, Hanwei Chen, Yanchun Zhu, Zijun Wei, Shi Su, Fei Yan, Bensheng Qiu, Xin Liu, Zhaoyang Fan

**Affiliations:** 1 University of Science and Technology of China, Hefei, China; 2 Shenzhen Institutes of Advanced Technology, Shenzhen, China; 3 Guangzhou Panyu Central Hospital, Guangzhou, China; 4 Cedars-Sinai Medical Center, Los Angeles, CA USA

## Background

3D self-gated (SG) cine imaging with TrueFISP not only provides excellent contrast between myocardium and blood, but also eliminates the need for ECG set up and permits free-breathing acquisitions [1]. However, such Cartesian sampling-based techniques are commonly used at 1.5 T due to the eddy current and SAR problems as well as time-consuming on data acquisition under the Nyquist sampling criteria. To achieve time-efficient 3T cine imaging, a novel accelerated SG method, named SparseSG, was proposed using a tiny golden angle and compressed sensing [2].

## Methods: Sequence

**Figure 1:**
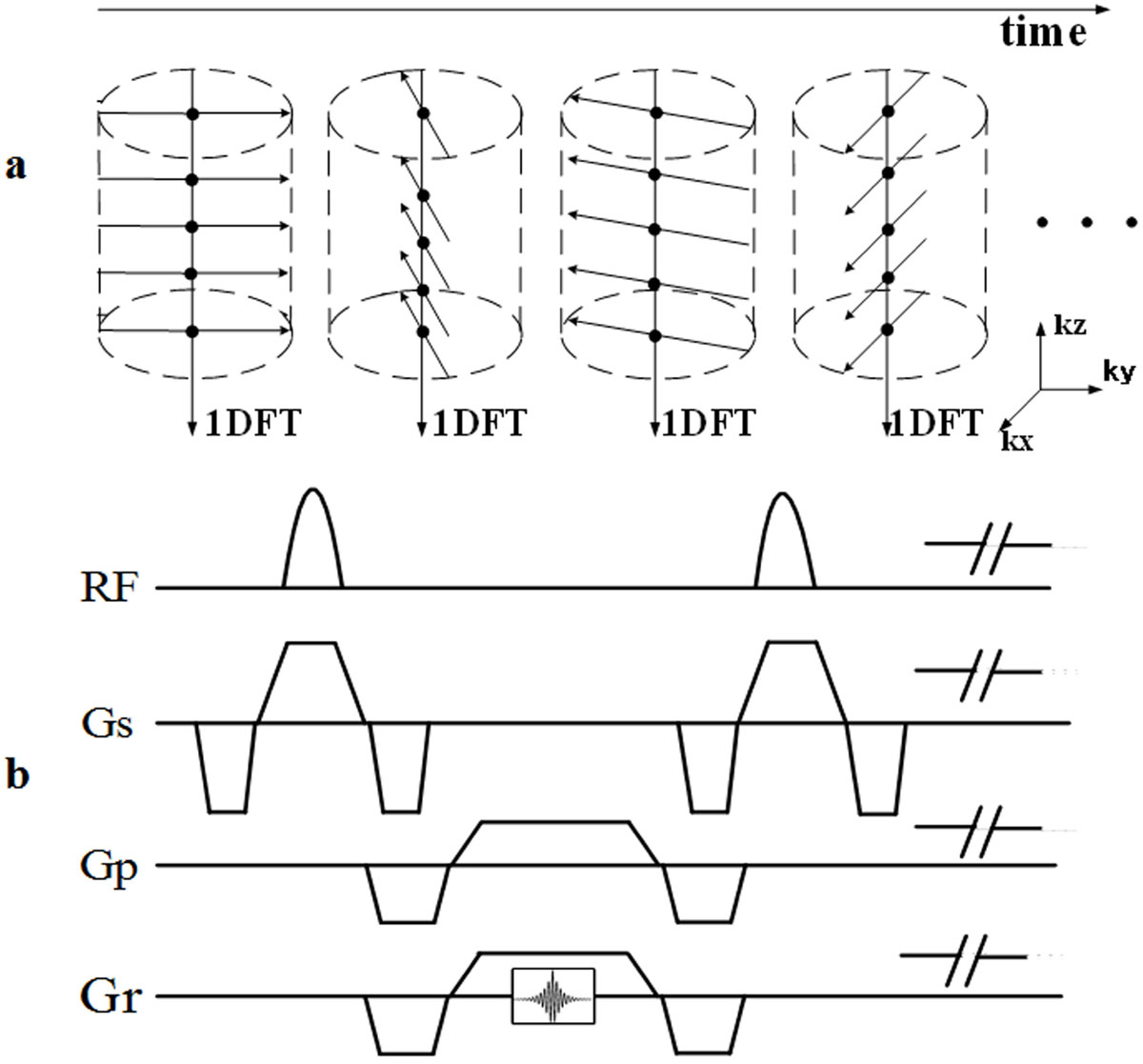
**Diagram of the proposed SparseSG technique**. (a) A tiny golden angle of 32.039°, instead of 111.246°, was used in the stack-of-stars sampling trajectories, the dot (•) denotes the SG data.(b) the diagram of the TrueFISP sequence with stack-of-stars sampling trajectories.

A 3D hybrid radial sampling pattern was adopted for the SparseSG [1]. In order to reduce the eddy current effect, a tiny golden angle of 32.039°, instead of 111.246°, was used for data acquisition (Figure 1). After the respiratory and cardiac motions were determined by processing the SG data as [1], the acquired data was retrospectively sorted into different respiratory and cardiac phases. A compressed sensing method exploiting the image sparsity in k-t space was used for image reconstruction, thus effectively shortening the scan time and reducing SAR.

## Experiment

IRB-approved cardiac imaging was performed on 5 healthy subjects (2M, 3F, age 20~26) at 3 T (Siemens Tim Trio, Germany) with a standard 6-channel body coil and a spine coil. Scan parameters included: 3D imaging with standard short-axis, TR = 3.8 ms, TE = 1.9 ms, spatial resolution = 1.3 × 1.3 × 8.0 mm^3^, bandwidth = 1502 Hz/Pixel, partition number = 10. The acceleration factors were R = 4 and 8, corresponding to scan time 0.76 min and 0.38 min. The standard 2D multi-slice ECG-triggering and conventional self-gating methods with the same spatial and temporal resolutions were also conducted for comparison.

## Results

**Figure 2:**
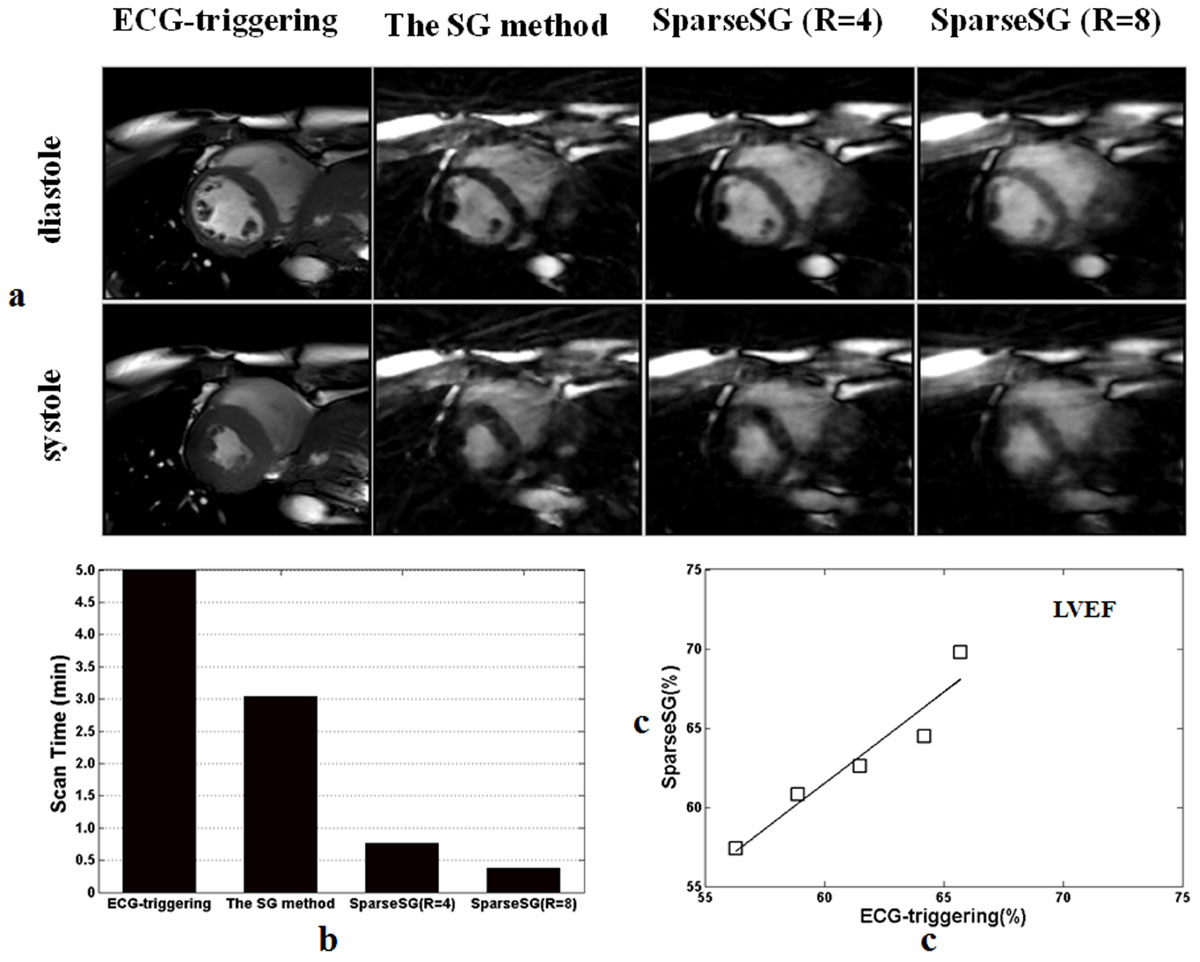
**(a) Representative images of the diastole (top row) and systole phases (bottom row) on SA view from a subject**. Compared to the standard ECG-triggering and conventional SG methods, cardiac structure obtained by SparseSG were well agreement with those by standard ECG-triggering and conventional SG methods. (b) Whole heart coverage of 3D cine imaging can be achieved within 1 min by SparseSG, which is much shorter than the standard ECG-triggering and conventional SG methods.(c) The left ventricular ejection fraction calculated from the images obtained by SparseSG was well agreement to the standard ECG-triggering method.

All MR scans were successfully conducted. SparseSG allowed a whole-heart coverage of 3D cine imaging within 1 min, which was much shorter than those of the ECG-triggering and SG methods (Figure 2.b). As the acceleration factor increased, the reconstructed cine images of SparseSG become a little blurry. However, the left ventricle ejection fraction (LVEF) and cardiac structure obtained from SparseSG were in good agreement with those from conventional ECG-triggering and SG methods, even if a high acceleration factor R = 8 was used (Figure 2.a&c).

## Conclusions

An accelerated SG technique, SparseSG, was developed to realize 3D cardiac cine imaging at 3T without ECG and breath-holding. Preliminary in vivo study demonstrated that whole heart coverage of 3D cine imaging can be achieved within 1 min and the technique has excellent performance compared to the standard ECG-triggering and conventional SG methods. This warrants further evaluation of SparseSG on more volunteers and patients.
